# Supplementary material for: Assessment of Minimal Residual Disease by Next Generation Sequencing in Peripheral Blood as a Complementary Tool for Personalized Transplant Monitoring in Myeloid Neoplasms
Source: J Clin Med. 2020 Nov 25;9(12):3818. doi: 10.3390/jcm9123818 (PMC7760908; doi:10.3390/jcm9123818)
Supplement: Supplementary file 1 [file jcm-09-03818-s001.pdf]

**Supplementary Materials:**

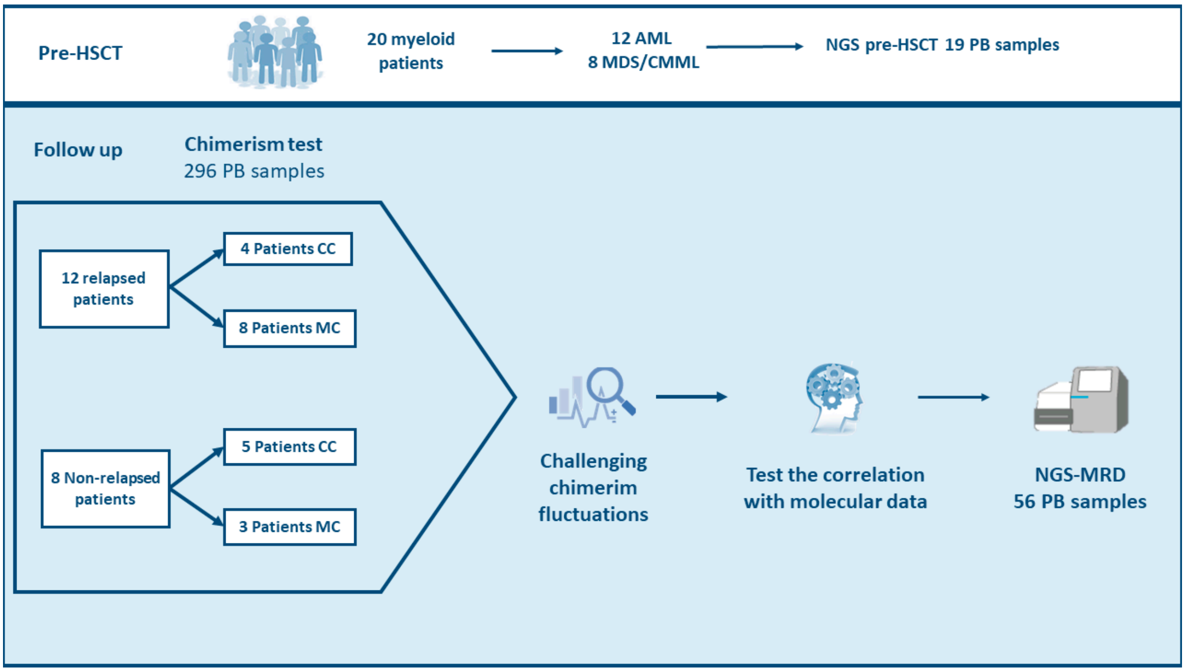


**Figure S1.** Flow-chart showing a description of patients and samples selection. We selected 20 patients (12 AML, 8 MDS/CMML) following allogenic hematopoietic stem cell transplant (HSCT). NGS was performed in 19 PB samples pre-HSCT. During the follow up, 296 PB DNA samples were analyzed by indel-qPCR and from those 56 PB samples were selected for NGS analysis when chimerism fluctuations were detected or to study the correlations with chimerism. After HSCT, patients were classified according to relapse: patients who relapsed after HSCT (n=12) and patients without relapse at the end of study (n=8). Both groups include patients achieving CC at some point during the follow up and patients with always MC.

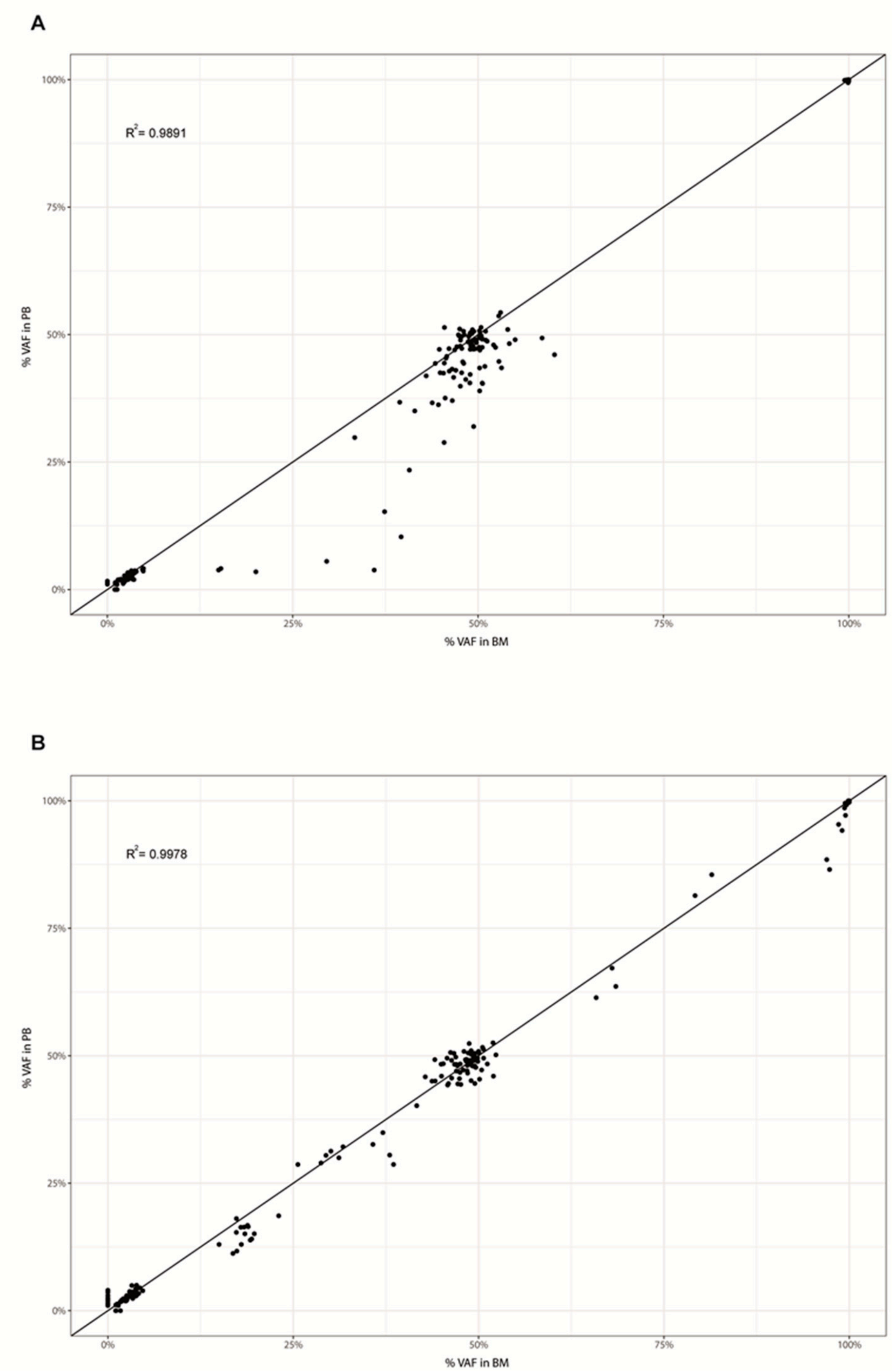


**Figure S2.** Correlation analysis of VAF percentage in peripheral blood and bone marrow-paired samples performed with Pearson correlation test. VAF comparison detected with the Pan-Myeloid Panel showed a high correlation of the 8 paired samples, including 4 samples at diagnosis $R^2$=0.9891, *p*-value < 0.0001 (A) and 4 samples at follow-up $R^2$=0.9978, *p*-value < 0.0001 (B) (VAF= Variant allele frequency; PB=Peripheral Blood; BM=Bone Marrow).

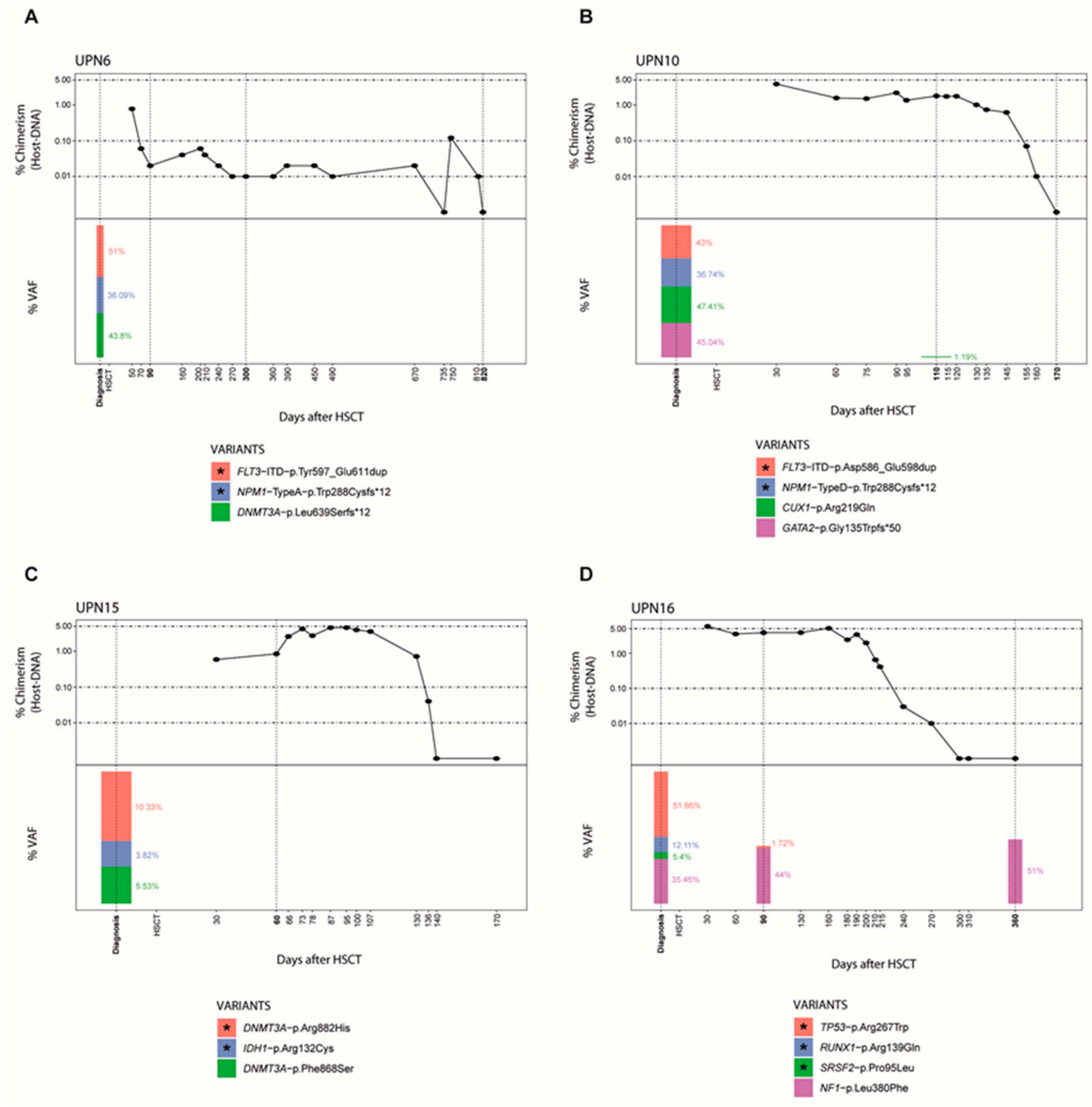


**Figure S3.** NGS analysis in non-relapsed patients achieving CC. Non-relapsed patients showed a correlation of chimerism status and NGS-MRD during the monitoring of the disease course for patients UPN6 (A), UPN10 (B), UPN15 (C), UPN16 (D) with clearance of all variants; only UPN16 showed a VUS confirmed in his sibling-donor. Post-HSCT engraftment analysis by indel-qPCR results are plotted as percentage of receptor (*Y*-axis) over time shown as days post-HSCT (*X*-axis). Vertical lines denote the NGS-analysis time points and the height bars represents VAF percentages of the pathogenic and likely pathogenic variants. (NGS=Next Generation Sequencing; MRD=Minimal Residual Disease; MC=Mixed Chimerism; CC=Complete Chimerism; HSCT=Hematopoietic Stem Cell Transplant; UPN=Unique Patient Number; VAF=Variant Allele Frequency).

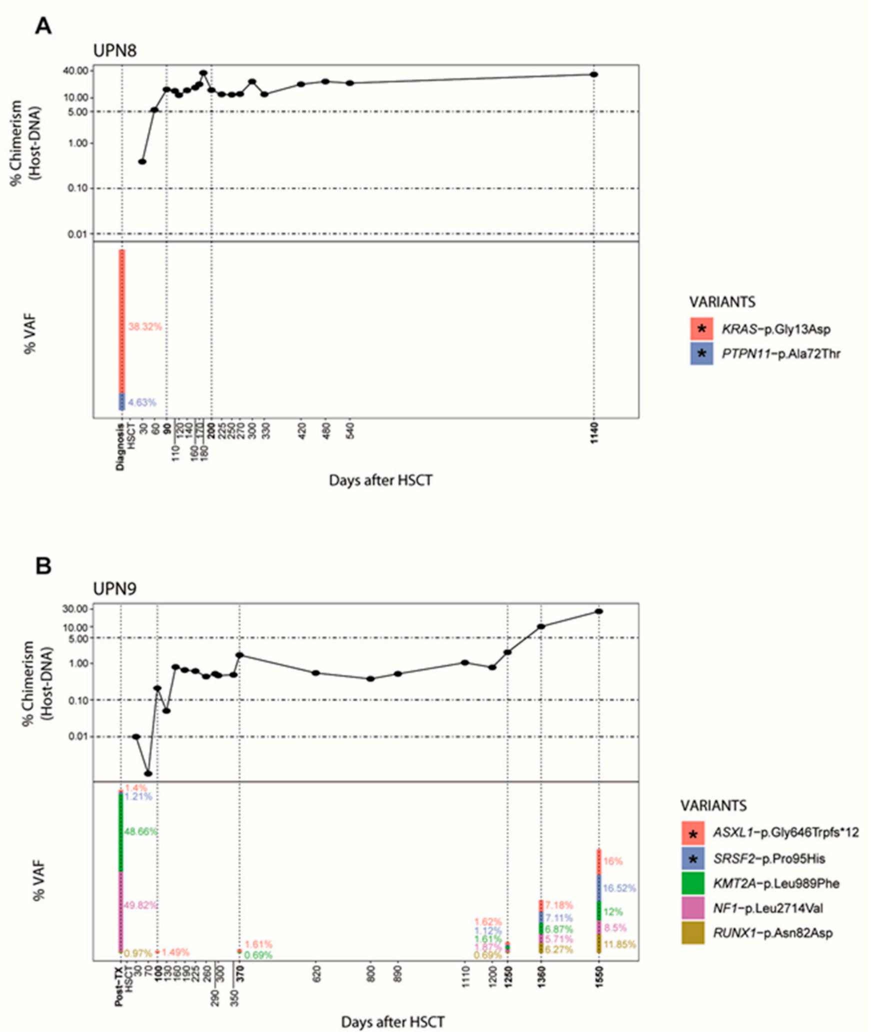


**Figure S4.** NGS analysis in non-relapsed patients with MC. Non-relapsed patients showed negative NGS-MRD despite presence of MC for patients UPN8 (A) and UPN9 (B). Post-HSCT engraftment analysis by indel-qPCR results are plotted as percentage of receptor (*Y*-axis) over time shown as days post-HSCT (*X*-axis). Vertical lines denote the NGS-analysis time points and the height bars represents VAF percentages of the pathogenic and likely pathogenic variants. (NGS=Next Generation Sequencing; MRD=Minimal Residual Disease; MC=Mixed Chimerism; CC=Complete Chimerism; HSCT=Hematopoietic Stem Cell Transplant; UPN=Unique Patient Number; VAF=Variant Allele Frequency).

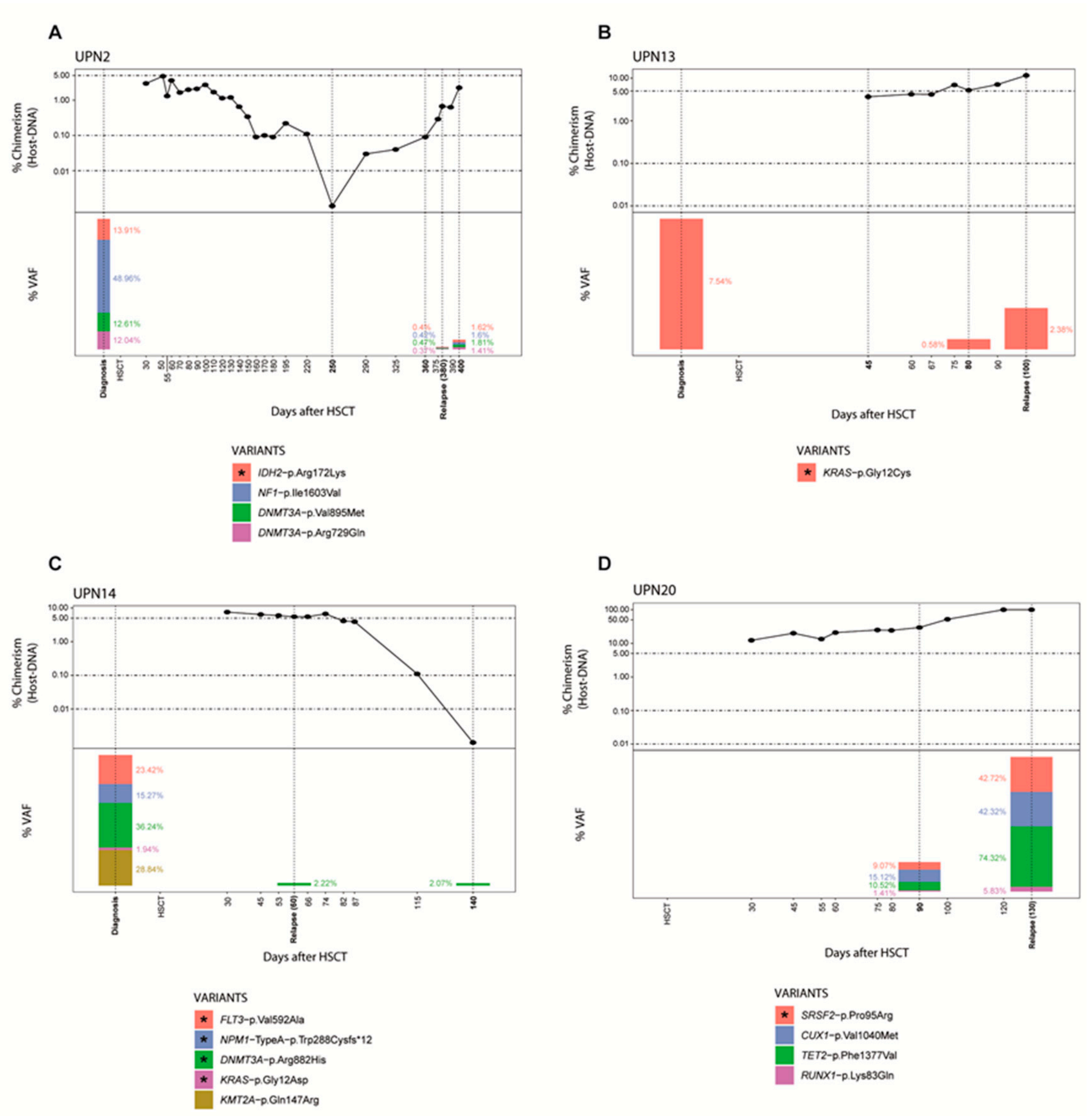


**Figure S5.** Relapsed patients with positive NGS-MRD. Positive NGS-MRD is detected at relapse of patients UPN2 (A), UPN13 (B), UPN14 (C), UPN20 (D). Post-HSCT engraftment analysis by indel-qPCR results are plotted as percentage of receptor (*Y*-axis) over time shown as days post-HSCT (*X*-axis). Vertical lines denote the NGS-analysis time points and the height bars represents VAF percentages of the pathogenic and likely pathogenic variants. (NGS=Next Generation Sequencing; MRD=Minimal Residual Disease; MC=Mixed Chimerism; CC=Complete Chimerism; HSCT=Hematopoietic Stem Cell Transplant; UPN=Unique Patient Number; VAF=Variant Allele Frequency).

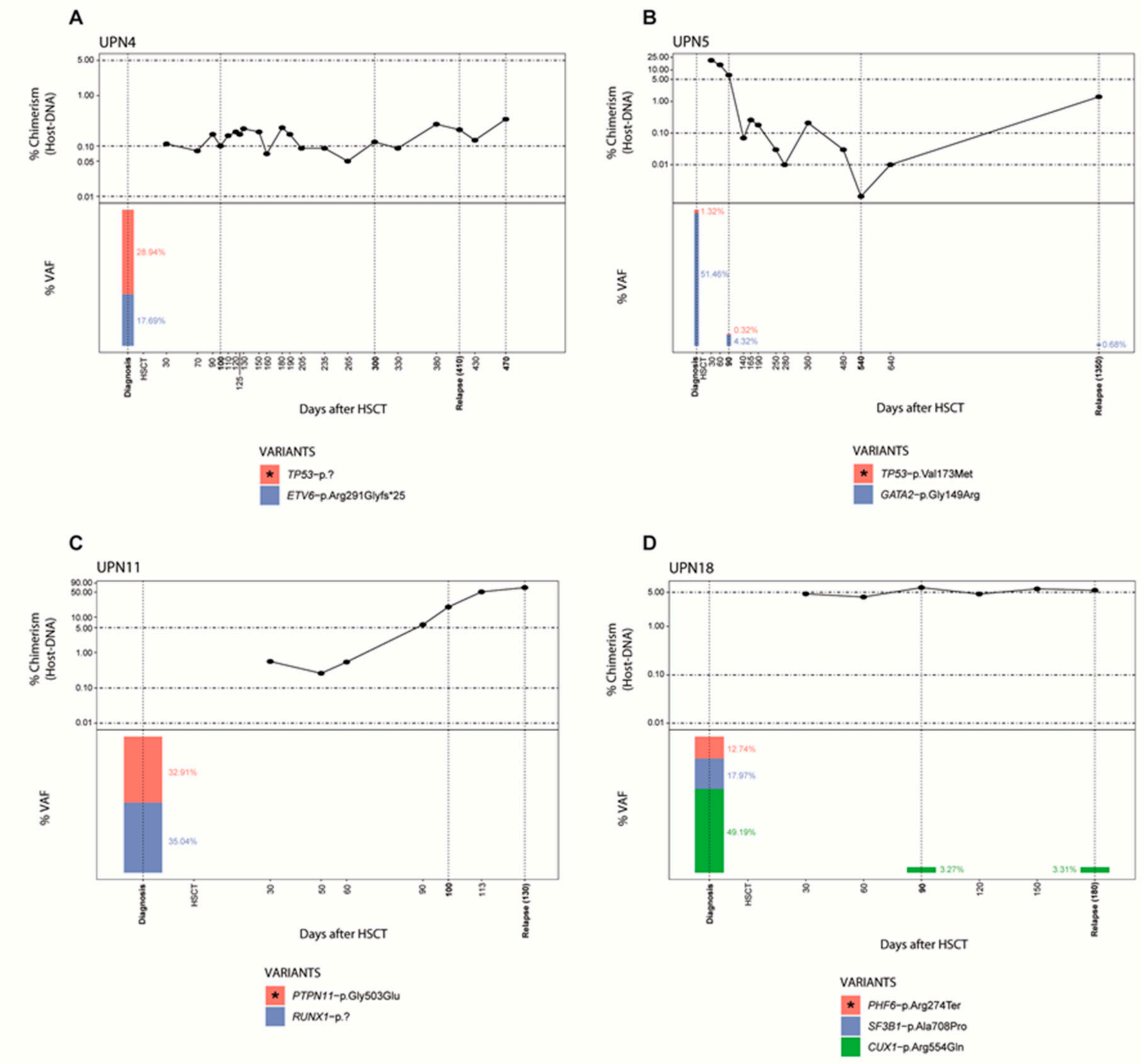


**Figure S6.** Relapsed patients with no positive NGS-MRD. No NGS-MRD variants were detected at the time of relapse for UPN5 (A), UPN4 (B), UPN11 (C), UPN18 (D). Post-HSCT engraftment analysis by indel-qPCR results are plotted as percentage of receptor (*Y*-axis) over time shown as days post-HSCT (*X*-axis). Vertical lines denote the NGS-analysis time points and the height bars represents VAF percentages of the pathogenic and likely pathogenic variants. (NGS=Next Generation Sequencing; MRD=Minimal Residual Disease; MC=Mixed Chimerism; CC=Complete Chimerism; HSCT=Hematopoietic Stem Cell Transplant; UPN=Unique Patient Number; VAF=Variant Allele Frequency).
